# An Interleaved Segmented Spectrum Analysis: A Measurement Technique for System Frequency Response and Fault Detection

**DOI:** 10.3390/s22186757

**Published:** 2022-09-07

**Authors:** Alejandro Roman-Loera, Anurag Veerabathini, Jorge E. Macias-Diaz, Felipe de Jesus Rizo-Diaz

**Affiliations:** 1 Department of Electronic Systems, Universidad Autónoma de Aguascalientes, Aguascalientes 20100, Mexico; 2 Department of Electrical & Computer Engineering, New Mexico State University, Las Cruces, NM 88003, USA; 3 Department of Mathematics and Didactics of Mathematics, School of Digital Technologies, Tallinn University, Narva Rd. 25, 10120 Tallinn, Estonia; 4 Department of Mathematics and Physics, Universidad Autónoma de Aguascalientes, Aguascalientes 20100, Mexico

**Keywords:** frequency response, sinc function, fast Fourier transform, digital signal processing, windowing technique, network analyzer, poles and zeros, Bode diagram, system stability

## Abstract

A frequency spectrum segmentation methodology is proposed to extract the frequency response of circuits and systems with high resolution and low distortion over a wide frequency range. A high resolution is achieved by implementing a modified Dirichlet function (MDF) configured for multi-tone excitation signals. Low distortion is attained by limiting or avoiding spectral leakage and interference into the frequency spectrum of interest. The use of a window function allowed for further reduction in distortion by suppressing system-induced oscillations that can cause severe interference while acquiring signals. This proposed segmentation methodology with the MDF generates an interleaved frequency spectrum segment that can be used to measure the frequency response of the system and can be represented in a Bode and Nyquist plot. The ability to simulate and measure the frequency response of the circuit and system without expensive network analyzers provides good stability coverage for reliable fault detection and failure avoidance. The proposed methodology is validated with both simulation and hardware.

## 1. Introduction

Recent modern automobiles are equipped with electronic systems with improved capabilities and safety features, such as an advanced driver-assistance system (ADAS) and a collision avoidance system for the protection of pedestrians and people inside the car. These systems use multiple sensors, such as cameras, proximity sensors, LIDAR, RADAR, microphones, transceivers, etc. Though sensed information is processed with a reliable microprocessor, the performance of these electronic devices depends on the analog circuits with which these sensor modules are built, such as amplifiers, data converters, power management circuits, and other signal processing and conditioning circuits. These analog circuits have a definitive frequency response whose stability is typically guaranteed by their phase margin and gain margin [1]. Limited frequency response tests and validation by approximating the system model in the simulation or by incorrect test procedures can cause the system to fail in the application field, and a failure of critical sensors or actuators, such as an airbag actuator, can be catastrophic. Typically, these circuits are required to operate in harsh environments over a wide temperature range [2]. Hence, it is crucial to understand the circuit behavior over a well-defined frequency range to ensure proper device operation.

The frequency response of a circuit and system is acquired by: (a) analyzing the transfer function (TF) using a linear model [1], (b) simulating either the actual system or its average model [3], and (c) hardware measurement using instruments, such as network analyzers. The TF analysis uses linear time-invariant system (LTI) methods and typically yields a complex equation with multiple poles and zeros, making it difficult to utilize for system improvement. The closed-form TF is simplified for better understanding and allows the designer to improve the system performance through increased bandwidth and phase margin. This simplification is achieved by reducing the number of poles and zeros. This process eliminates a few weak dependency components, such as parasitics from the analysis. Ignoring a critical pole or zero during this simplification process causes a mismatch in the frequency response between the analysis and measurement, which can cause catastrophic failure in real-life applications. Hence, the TF can be used for system behavior only for a finite frequency range in which their responses match.

Some circuits or electrical machines cannot be represented as a TF due to their complexity. Multiple methodologies have been proposed in the literature [4,5,6] that use an equivalent model with system approximations to achieve the TF followed by additional simplifications. These methods apply to a limited frequency range, and additional tests are required to verify the TF and the system. Other approaches have been proposed [7,8] that approximate the linear TF to the behavior of nonlinear systems, such as switching the regulator circuits that operate with pulse width modulation (PWM) control schemes [9,10,11,12,13]. A frequency response analysis is used to estimate the power transformer and induction motor models [14,15,16,17,18,19,20,21]. However, the system frequency response and stability should be measured to ensure safe and correct operation to avoid any system faults and failures.

The frequency response is characterized under LTI systems [22,23,24] and is analyzed in the complex frequency domain, also known as the s-domain. Despite that, the theory of LTI systems can be transferred to the frequency domain due to the similarity between the definitions of the Laplace transform (LT) and Fourier transform (FT). The s-domain provides the stability of the system, whereas the frequency domain provides the frequency response of the system. Though they yield different information, some characteristics in the frequency domain can be observed from the response in the s-domain and vice versa. It is worth mentioning that the analysis in the s-domain provides a closed-form expression that cannot be used to measure the frequency response directly. Instead, transformation into the frequency domain allows for observation of the frequency response and acquisition of stability characteristics to detect failure conditions.

The most common method for extracting the frequency response is to apply a single-tone signal in the time domain to the input of the system under test and measure the signal transmitted to the output of the system. The amplitude ratio of the output-to-input signal provides the system gain. The phase difference between the input and output signals provides the system phase. This method tests the system one tone at a time, and frequency sweeping is required to get a frequency response from time-domain signals. Additionally, the frequency resolution depends on the number of tones swept. The proposed measurement method tests a specific number of tones at a time to decrease the measurement time. The resolution of the measurement is improved using the proposed method, where the frequency spectrum is divided into multiple segments with a high number of harmonic elements.

References [25,26,27] describe methods in which a dedicated signal generation circuit, such as a voltage-controlled oscillator, and magnitude and phase measurement circuits are implemented along with the design under test on the same integrated circuit (IC). These methods require the system under test to operate in a continuous-time (CT) domain, and the measurement suffers from high sensitivity to noise, which affects its precision and accuracy. Moreover, these methods use additional filtering on the signals, increasing the complexity of the circuits. To solve this issue, multiple approaches are proposed in the literature [8,28,29] to convert CT signals into discrete-time (DT) signals. These discrete signals are processed using digital signal processing (DSP) methods and translated to the frequency domain. The method reported in [28] uses cross-correlation to reconstruct the frequency response. The method proposed in [29] uses signal cross-correlation and the fast Fourier transform (FFT) to extract the frequency response of the system. In [8], the use of only a 64-component multi-tone signal for the entire frequency response with the FFT has caused a low-resolution frequency response. All of the proposed methods in the literature do not limit or prevent the FFT or spectral leakage that is observed when a real sinusoidal signal is used in the hardware. Moreover, all of the methods assume an ideal sinusoidal signal with a single-frequency tone that is limited to simulations. The spectral leakage adds a distortion in the frequency and causes inaccuracies in the frequency response measurement.

A frequency spectrum segmentation methodology for extracting the frequency responses of circuits and systems with a high resolution and low distortion over a wide frequency range is proposed. The proposed methodology can be both simulated and measured on hardware. A high resolution is achieved by implementing a modified Dirichlet function (MDF) configured for multi-tone excitation signals. Low distortion is attained by limiting or avoiding spectral leakage and interference into the frequency spectrum of interest. The windowing technique is popularly used in the literature to reduce interference. The proposed method uses a new windowing technique, called the double-frequency Hann window, for further reduction in the signal distortion and suppression of the system-induced oscillations that can cause severe interference while acquiring signals. The proposed window function pushes the undesired harmonic components generated by the window modulation to designated frequency locations, which reduces the spectral leakage introduced by the windowing technique. The proposed segmentation methodology together with the MDF generates an interleaved frequency spectrum segment. Different spectrum segments can be obtained by configuring the MDF, and these segments can be set up in a logarithmic frequency scale to measure the spectral content in frequency decades. Overlapping the spectral content of the different segments, the entire circuit or system frequency response can be reconstructed with a relatively high resolution and low distortion, and it can be represented in a Bode and Nyquist plot after post-processing. The ability to simulate and measure the frequency response of the circuit and system without expensive network analyzers helps to obtain good performance coverage for reliable fault detection and failure avoidance.

## 2. Theoretical Analysis

### 2.1. The Sinc Function

The sinc is a mathematical function widely used in DSP applications to explain the behavior of devices in the discrete-time domain. A monotonically decreasing function 1/x and an oscillating signal sin(xt) with a period of 2π are combined to form sinc(xt). As a result, sinc(xt) has sinusoidal oscillations with a period of 2π and an amplitude that continually decreases as 1/x. The frequency spectrum of a sinc function is a uniform magnitude with a fixed bandwidth. The definition in the time domain is given by:

(1) $$\mathrm{sinc}\left(xt\right)=\left\{\begin{array}{cc} \frac{\sin(xt)}{xt} & ;x\neq 0\\ \;1 & ;x=0 \end{array}\right.$$

where x=2πf is the frequency component of the sine function.

**Figure 1:**
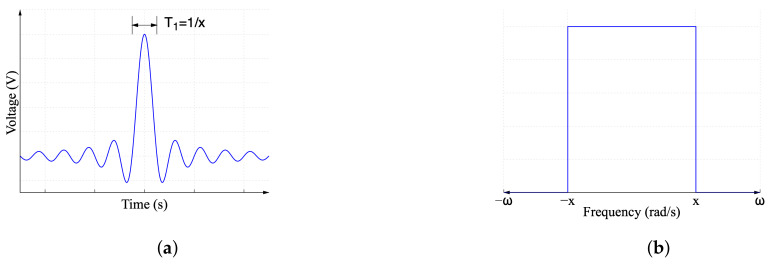
Sinc function in (**a**) time domain and (**b**) frequency domain (magnitude spectrum).

Time-domain signals are transformed into the frequency domain using the Fourier transform. The frequency spectrum of a sinc function is given as:

(2) $$\mathrm{sinc}\left(xt\right)\overset{F}{\to }F\left(j\omega \right)=\Pi \left(j\omega \right)=\left\{\begin{array}{cc} \frac{\pi }{x} & ;|\omega |<|x|\\ 0 & ;|\omega |>|x| \end{array}\right.$$

where Π(jω) represents the rectangular pulse in the frequency domain. The sinc function in Equation (1) in the time domain is shown in Figure 1a. The magnitude of the frequency spectrum described by Equation (2), showing the typical characteristics of the sinc function in the frequency domain, is shown in Figure 1b.

The signal described by Equation (1) helps in understanding the signal behavior, but its implementation with a continuous-time signal in the hardware is impractical. Digital signal generators with an arbitrary waveform generator (AWG) function provide a discrete-time approximation that preserves its key characteristics, but this causes undesired effects, which should be considered to minimize the interference and distortion.

**Figure 2:**
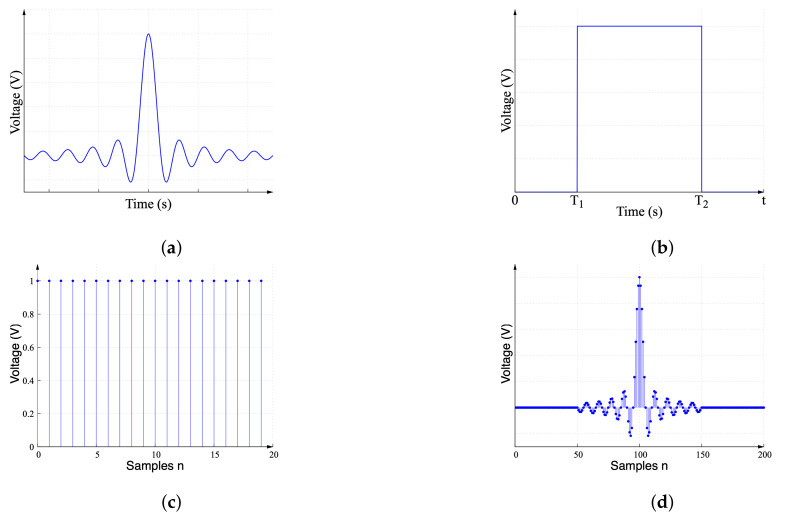
Sinc function generation showing (**a**) sinc function in continuous-time domain, (**b**) rectangular pulse in continuous-time domain, (**c**) unitary impulse train, and (**d**) practical discrete-time sinc function.

The discrete-time sinc function is generated by reducing the sinc function defined in −∞≤t≤∞ to a finite range shown in Figure 2a and multiplying it by its rectangular pulse shown in Figure 2b in the time domain. The finite duration continuous-time sinc signal is multiplied by the unitary impulse train shown in Figure 2c to generate the discrete-time sinc signal in Figure 2d. This discrete-time sinc signal can be generated using an AWG in hardware. This signal can be represented in the frequency domain by using the convolution operation. Consider that $S_{1}\left(j\omega \right)$ and $S_{2}\left(j\omega \right)$ are the Fourier transforms for the signals shown in Figure 2a and Figure 2b, respectively, and are given by:

(3) $$S_{1}\left(j\omega \right)=F\left\{\mathrm{sinc}\left(\omega t\right)\right\},$$

(4) $$S_{2}\left(j\omega \right)=F\left\{\Pi \left(kt\right)\right\},$$

(5) $$S_{3}\left(j\omega \right)=S_{1}\left(j\omega \right)*S_{2}\left(j\omega \right),$$

where $S_{3}\left(j\omega \right)$ is the convolution between $S_{1}\left(j\omega \right)$ and $S_{2}\left(j\omega \right)$.

**Figure 3:**
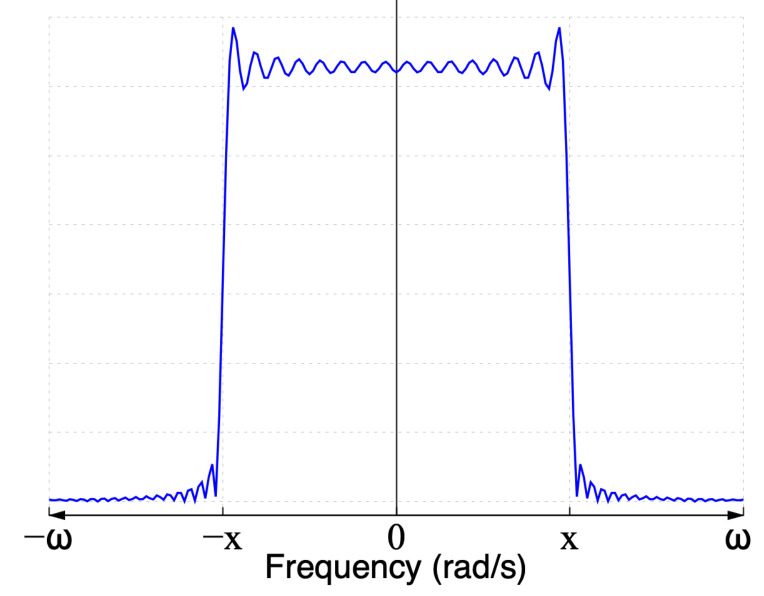
Magnitude spectrum of a sinc function limited in time.

The closed-form signal definition from the convolution in Equation (5) in terms of the sine integral in the frequency domain is given by [30]:

(6) $$Si\left(x\right)=\int_{0}^{x}\frac{\sin(t)}{t}dt$$

where Si(jω) represents the frequency spectrum of a sinc function limited in time, and its spectrum is shown in Figure 3. Observe that there are differences compared to an ideal sinc frequency spectrum. The edges are not as sharp as in Figure 1b, and the magnitude spectrum is not as uniform as shown in Figure 1b. The oscillations observed in Figure 3 are described in the literature as the Gibbs effect [31,32], and they are caused by the limited time duration of the ideal sinc function.

The Fourier transform of the sampled sinc function of Figure 2d is obtained by the convolution of $S_{3}\left(j\omega \right)$ and the Fourier transform of the impulse train shown in Figure 2c. The Fourier transform of the impulse train in the time domain is another impulse train in the frequency domain [32] given by

(7) $$S_{4}\left(j\omega \right)=\sum_{k=-\infty }^{\infty }\delta \left(j\left(\omega +k\Omega_{s}\right)\right).$$

where $\Omega_{s}$ is the sampling frequency given by $\Omega_{s}=\frac{2\pi }{T_{s}}$, and $T_{s}$ is the sampling time. The Fourier transform of a practical sinc signal is given by:

(8) $$S_{5}\left(j\omega \right)=S_{3}\left(j\omega \right)*S_{4}\left(j\omega \right)=\sum_{k=-\infty }^{\infty }S_{3}\left(j\left(\omega +k\Omega_{s}\right)\right).$$

Observe that the frequency spectrum in Equation (8) is replicated every $k\Omega_{s}$ due to aliasing [31,32].

**Figure 4:**
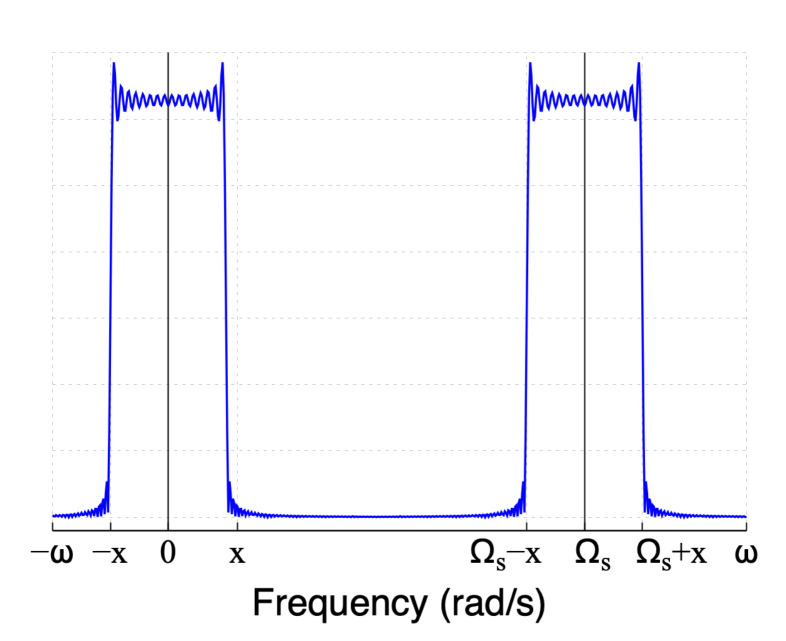
Magnitude spectrum of the practical sinc function.

The magnitude of the frequency spectrum of the practical sinc function is shown in Figure 4, where the frequency spectrum is replicated as aliasing with a central frequency of $k\Omega_{s}$, for k=0,1,2, and so on. Although a practical sinc signal has a bandwidth similar to an ideal sinc function, its usage requires several considerations to minimize the impact of unwanted aliasing and the Gibbs effects. Aliasing is controlled by limiting the signal bandwidth using low-pass filtering and by defining the sampling time $T_{s}$. Limiting and avoiding the unwanted Gibbs effect is challenging and depends on the application of the signal. In FIR filters, the use of the windowing technique helps reduce the Gibbs effect but not completely. Applying the windowing technique for practical sinc signal application distorts the measurements instead of reducing the Gibbs effect.

### 2.2. The Dirichlet Function and the Modified Dirichlet Function

Sharp edges are required in the frequency spectrum for data transmission applications. Multiple methods are proposed in the literature to achieve sharp edges for a sinc signal in discrete time. For instance, [33,34] proposed sinc-shaped Nyquist pulses in which a periodic sinc comb, also called the Dirichlet function (DF) [35], is used, and it is given by:

(9) DN(t)=sin(NΩ1t)Nsin(Ω1t);Ω1t≠πk,kk=0,1,2,3,…(−1)k(N−1);Ω1t=πk,kk=0,1,2,3,…

where $\Omega_{1}$ is the frequency of separation between the spectrum components, and $N\Omega_{1}$ is the spectrum bandwidth.

**Figure 5:**
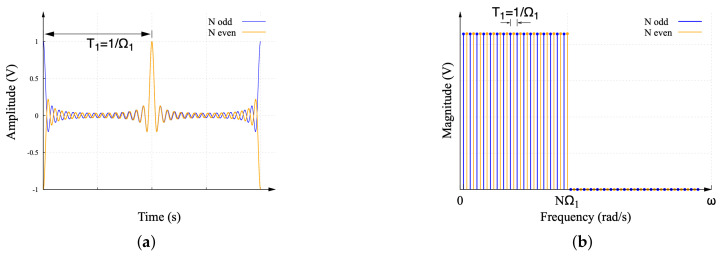
(**a**) Dirichlet function and its (**b**) Fourier transform.

The Dirichlet function in the time and frequency domains is shown in Figure 5. Observe that the Fourier transform of this function is a uniformly distributed pulse with a defined frequency and sharp edges. Our proposed work uses the Dirichlet function as a frequency-sampled alternative for the frequency spectrum of the ideal sinc function with a reduced Gibbs distortion. The DF has two components, as shown in Figure 5a. The first component is configured when *N* is odd, and the second component is configured when *N* is even in Equation (9). The frequency spectrum of these components contains all of the odd and even components of the equivalent ideal sinc function spectrum, as shown in Figure 5b. The combination of the frequency spectra of the two DF components configures the magnitude of an interleaved ideal sinc function spectrum. The zero-magnitude frequency components of these spectra provide an opportunity for the use of single-tone windowing with no distortion or leakage over the spectral components of interest.

A high number of zero-magnitude harmonic components is desired for the practical implementation of the proposed measurement technique. The number of zero-magnitude harmonic components between non-zero harmonic components can be increased with the proposed modified Dirichlet function (MDF) and is given by:

(10) RN(t)=sin(2NΩ1t)Nsin(2Ω1t)Ω1t≠πk,kk=0,±1,±2,±3,…(−1)k(N−1),Ω1t=πk,kk=0,±1,±2,±3,…

The proposed MDF $R_{N}\left(t\right)$ has four components, but only two components can be obtained from the closed-form equation in Equation (10). The first component is defined when *N* is odd, where the frequency spectrum of the MDF contains the frequency components $\left[2,6,10,\ldots ,N\Omega_{1}-2\right]$. The second component is defined when *N* is even, where the frequency spectrum contains the harmonic components $\left[4,8,12,\ldots ,N\Omega_{1}\right]$. The spectral content corresponding to odd components, $\left[1,5,9,\ldots ,N\Omega_{1}-3\right]$ and $\left[3,7,11,\ldots ,N\Omega_{1}-1\right]$ is missing, and it cannot be defined by the closed-form expression of $R_{N}\left(t\right)$.

Referring to Equation (10) which is a continuous signal, the extracted discrete spectral contents of each component sampled at N/4 in MDF from the simulation are given by:

(11) $$R_{1_{N}}\left(t\right)=\sum_{k=1}^{\frac{N}{4}}\frac{4\cos(2*N\left(2k-1\right)\Omega_{1}t)}{N},$$

(12) $$R_{2_{N}}\left(t\right)=\sum_{k=1}^{\frac{N}{4}}\frac{-4\cos(N\left(4k-3\right)\Omega_{1}t)}{N},$$

(13) $$R_{3_{N}}\left(t\right)=\sum_{k=1}^{\frac{N}{4}}\frac{-4\cos(N\left(4k-1\right)\Omega_{1}t)}{N},$$

(14) $$R_{4_{N}}\left(t\right)=\sum_{k=1}^{\frac{N}{4}}\frac{4\cos(N4k\Omega_{1}t)}{N},$$

where $R_{1_{N}}$ and $R_{4_{N}}$ add up to be all of the even frequency components of an ideal sinc function, and $R_{2_{N}}$ and $R_{3_{N}}$ in conjunction contain the odd frequency components.

**Figure 6:**
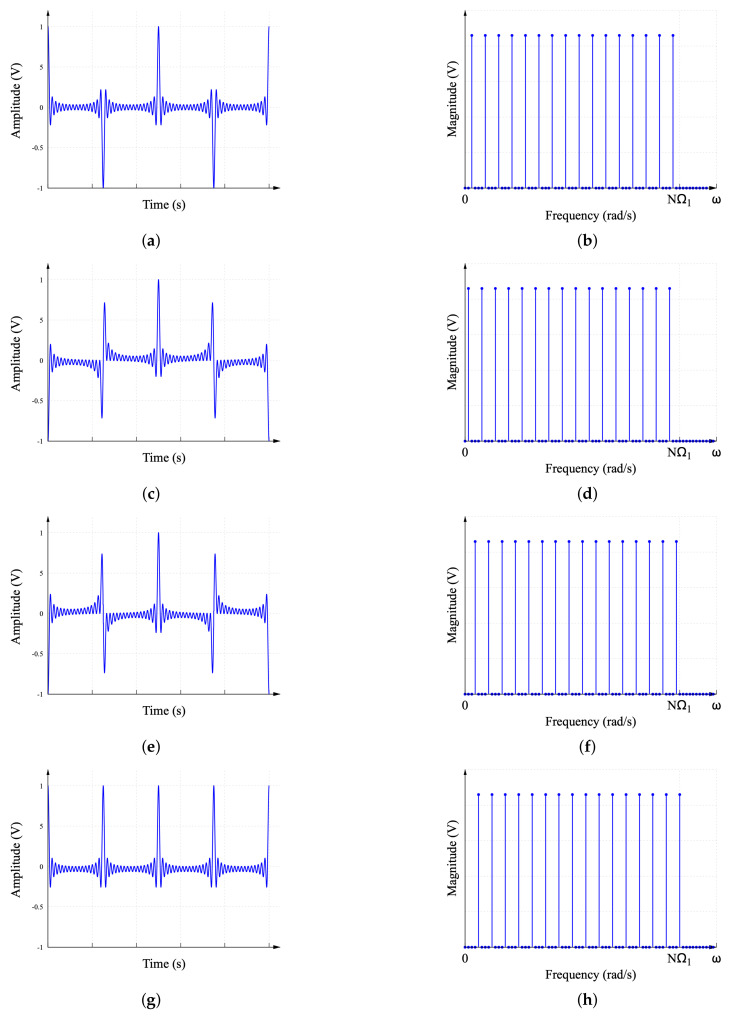
MDF waveforms. (**a**) First component of the MDF $R_{1_{N}}\left(t\right)$. (**b**) Magnitude of frequency spectrum $R_{1_{N}}\left(j\omega \right)$. (**c**) Second component of the MDF $R_{2_{N}}\left(t\right)$. (**d**) Magnitude of frequency spectrum $R_{2_{N}}\left(j\omega \right)$. (**e**) Third component of the MDF $R_{3_{N}}\left(t\right)$. (**f**) Magnitude of frequency spectrum $R_{3_{N}}\left(j\omega \right)$. (**g**) Fourth component of the MDF $R_{4_{N}}\left(t\right)$. (**h**) Magnitude of frequency spectrum $R_{4_{N}}\left(j\omega \right)$.

Figure 6 shows the waveform of the four components described by the MDF. In turn, Figure 6a corresponds to the waveform of $R_{1_{N}}\left(t\right)$. Observe in Figure 6b that $R_{1_{N}}\left(j\omega \right)$ contains the frequency components $\left[2,6,10,\ldots ,N\Omega_{1}-2\right]$. The waveforms of $R_{2_{N}}\left(t\right)$$R_{3_{N}}\left(t\right)$, and $R_{4_{N}}\left(t\right)$ are presented in Figure 6c, Figure 6e, and Figure 6g, respectively. The spectral content can be found in Figure 6d, Figure 6f, and Figure 6h, where $R_{2_{N}}$, $R_{3_{N}}$, and $R_{4_{N}}$ contain the frequency components $\left[1,5,9,\ldots ,N\Omega_{1}-3\right]$, $\left[3,7,11,\ldots ,N\Omega_{1}-1\right]$, and $\left[4,8,12,\ldots ,N\Omega_{1}\right]$, respectively. Comparing the MDF with respect to the original DF, it can be seen in Figure 5b that the DF contains only one zero-magnitude harmonic component between the non-zero harmonic components, whereas the MDF spectra in Figure 6b,d,f,h contain three zero-magnitude harmonic components between the non-zero harmonic components. The purpose of these free spaces in the spectra is discussed later.

**Figure 7:**
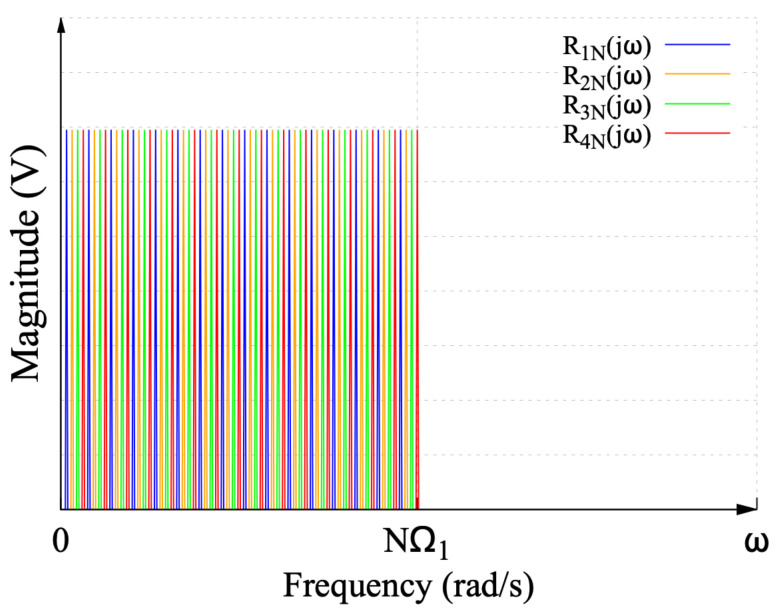
Frequency spectrum of the ideal sinc function recovered from the interleaved spectra of the modified DF.

Similar to the DF spectra, the combination of the four MDF spectra in Figure 6b,d,f,h configures an interleaved frequency spectrum of an ideal sinc function. This interleaved spectrum is shown in Figure 7.

### 2.3. The MDF and LTI Systems

**Figure 8:**
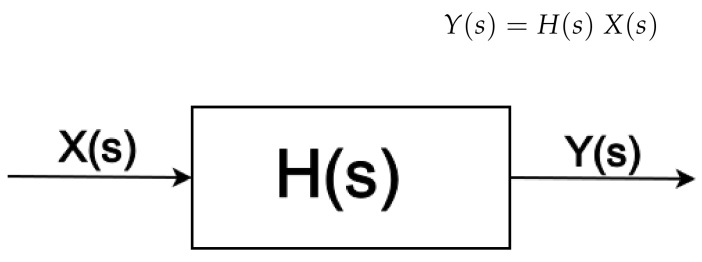
Block diagram of a system in s-domain.

The output signal of an LTI system Y(s) with a transfer function H(s) in the s-domain and the input signal X(s) shown in Figure 8 are given by:

(15) Y(s)=H(s)X(s)

As described earlier in Section 1, the Laplace transform provides information on the stability of the system, whereas the Fourier transform provides the frequency response. The Laplace transform can be used to obtain a mathematical closed-form equation that cannot be measured. On the other hand, the Fourier transform can be used to describe and measure the system frequency response. As the frequency response of the system can be measured by observing the input and output signals, its transfer function can be obtained by their ratio using Equation (15):

(16) $$H\left(j\omega \right)=\frac{Y(j\omega )}{X(j\omega )}$$

where H(jω) is the frequency response of the system, and Y(jω) and X(jω) are the Fourier transforms of the input and output signals, respectively. The magnitude of the complex number H(jω) corresponds to the magnitude spectrum of the frequency response of the system, whereas the phase of H(jω) is the phase shift between the input and output signals. The frequency response of the system can be reconstructed simply by observing the Fourier transform of the input and output signals.

The measurement of the frequency response of the system with one MDF requires high computational resources and becomes challenging. When the proposed technique is implemented in simulation, a high number of frequency components and a long FFT can cause the simulation to slow down and sometimes break. Similar challenges are observed during hardware implementation. Moreover, one MDF frequency measurement has a lower resolution, and it is discussed in the next section. A spectrum segmentation approach is proposed to allow the implementation of the proposed MDF technique in hardware and software.

## 3. Proposed Technique

### 3.1. Spectrum Segmentation

The spectrum segmentation consists of adjusting an MDF with the spectral content required to measure a frequency decade to the resolution required. The measurement of the different segments will configure the complete frequency response. The spectrum segmentation allows the achievement of a uniform resolution over all the frequency spectrum measurements. The MDF used to obtain different spectrum segments can be configured to provide a constant resolution per decade. A Bode plot is typically used for the frequency response with the frequency represented in a logarithmic scale using octave and decade segments. The proposed technique describes the segmentation of decades.

The discrete-time Fourier transform (DTFT) is the Fourier transform of a discrete-time signal whose output is continuous in frequency and periodic. However, the DTFT cannot be implemented in hardware directly. Alternatively, the discrete Fourier transform (DFT), which is a sampled version of the DTFT in the frequency domain, can be used for implementation. The magnitude and phase spectra obtained from the DFT are reconstructed using discrete-frequency components known as bins. The DFT is computed using the FFT, which is an efficient computational algorithm to obtain the DFT.

Frequency leakage occurs when any frequency component in the signal cannot be represented by a single bin and uses a combination between neighboring bins. Frequency leakage is one of the critical distortions in the frequency spectra obtained through the DFT. To reduce frequency leakage, the MDF signals should be configured in such way that the spectral content of any MDF components must find a bin contained in the FFT. This is limited to simulation, as an ideal sinusoidal signal with an accurate frequency cannot be generated with a waveform generator instrument. Hence, a long separation between the non-zero components in the spectral content is used to allow moderate leakage in the frequency components without causing interference to the other non-zero frequency components.

The proposed spectrum segmentation depends on the resolution of the frequency response measurement. If the spectral content of the MDF has three decades of width, a resolution as low as 10 points/decade is achieved for the first decade. The second and third decades will have a resolution of 90 points/decade and 900 points/decade, respectively. Thus, significant signal information is lost for the first decade, and a precise frequency measurement can be achieved in the third decade of the spectrum segment.

Consider a frequency measurement in the range from 100 Hz to 1 kHz with a resolution of 900 points/decade. The MDF bandwidth used to drive the LTI system requires 1000 frequency components, of which 900 components correspond to the desired 100–1000 Hz, 90 to 10–100 Hz, and 10 to 1–10 Hz. Therefore, the MDF should include three decades of spectral content with 1000 tones for 1 Hz bin frequency. In Figure 6, a system with $\Omega_{1}=2\pi \left(1\;\mathrm{Hz}\right)=2\pi$ rads/s and N=1000 for Equations (11)–(14) is given by:

$$R_{1_{1000}}\left(t\right)=\sum_{k=1}^{250}\frac{\cos(2000\left(2k-1\right)\Omega_{1}t)}{250},$$

$$R_{2_{1000}}\left(t\right)=\sum_{k=1}^{250}\frac{-\cos(1000\left(4k-3\right)\Omega_{1}t)}{250},$$

$$R_{3_{1000}}\left(t\right)=\sum_{k=1}^{250}\frac{-\cos(1000\left(4k-1\right)\Omega_{1}t)}{250},$$

$$R_{4_{1000}}\left(t\right)=\sum_{k=1}^{250}\frac{\cos(4000k\Omega_{1}t)}{250}.$$

**Figure 9:**
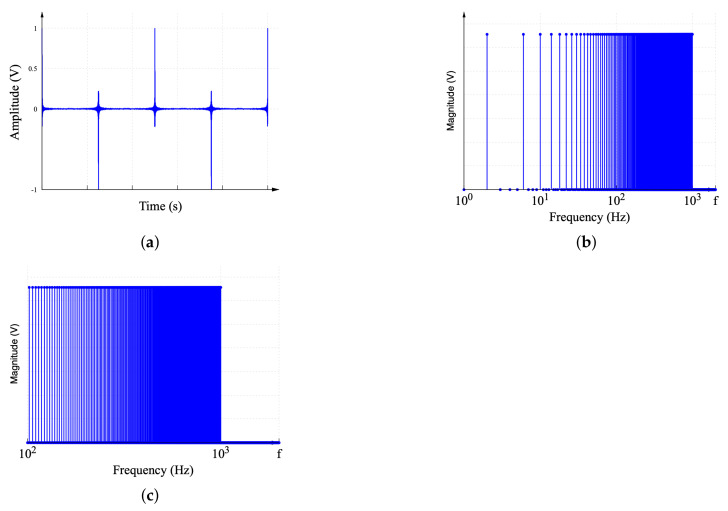
Characterization of 100–1000 Hz spectrum segment. (**a**) MDF configured to measure the spectrum segment of interest. (**b**) DFT of the MDF. (**c**) Segmented spectrum.

The above signals can be both simulated and generated using a waveform generator instrument. These continuous-time MDF signals are further required to be translated into discrete-time and are given by

$$R_{1_{1000}}\left[nT\right]=\sum_{k=1}^{250}\frac{\cos(2000\left(2k-1\right)\Omega_{1}nT)}{250},$$

$$R_{2_{1000}}\left[nT\right]=\sum_{k=1}^{250}\frac{-\cos(1000\left(4k-3\right)\Omega_{1}nT)}{250},$$

$$R_{3_{1000}}\left[nT\right]=\sum_{k=1}^{250}\frac{-\cos(1000\left(4k-1\right)\Omega_{1}nT)}{250},$$

$$R_{4_{1000}}\left[nT\right]=\sum_{k=1}^{250}\frac{\cos(4000k\Omega_{1}nT)}{250},$$

where $R_{1_{1000}}\left[nT\right]$, $R_{2_{1000}}\left[nT\right]$, $R_{3_{1000}}\left[nT\right]$, and $R_{4_{1000}}\left[nT\right]$ are the discrete-time sequences that describe the $R_{1_{1000}}\left(t\right)$, $R_{2_{1000}}\left(t\right)$, $R_{3_{1000}}\left(t\right)$, and $R_{4_{1000}}\left(t\right)$ components of the MDF, respectively. Here, *T* is the sampling time, and the sampling frequency is $F_{s}=\frac{1}{T}$. The sampling time should be selected considering the highest frequency component in the MDF and must meet the Nyquist criterion. In this case, to reduce the distortion in the 1 kHz signal and to improve the FFT computation, the sampling frequency $F_{s}=65.536\;\mathrm{kHz}$ is considered. This provides $2^{N}$ factor samples. The resulting signal and its spectrum are shown in Figure 9.

A plot of the discrete-time $R_{1_{1000}}\left[nT\right]$ sequence is shown in Figure 9a. The magnitude of the frequency spectrum of $R_{1_{1000}}\left[nT\right]$, obtained by computing the FFT of the sequence shown in Figure 9a, is presented in Figure 9b. The third decade of the spectrum, in Figure 9b, contains higher frequency components and is used to characterize the spectrum segment of 100–1000 Hz, as shown in Figure 9c. The spectral content of Figure 9c was expected to have 900 frequency components, but it actually has 225. The other 675 components are obtained by repeating the process with the sequences $R_{2_{1000}}\left[nT\right]$, $R_{3_{1000}}\left[nT\right]$, and $R_{4_{1000}}\left[nT\right]$. The full resolution of 900 points/decade is achieved when the MDF spectra are interleaved. This process should be repeated multiple times as necessary to complete the full frequency range of the frequency response.

### 3.2. Windowing Technique

The windowing technique is a popular DSP method used to reduce spectral leakage. Multiple window functions are proposed in the literature. However, it is worth pointing out that the selection of a specific window function depends on the FFT application, and the criterion of window selection is typically a trial-and-error process. In general, the windowing technique is used when there are low-frequency components compared to the bins in the spectral content that cause leakage over the frequency spectrum.

Since the MDF signal is configured to provide low leakage, the main source of the leakage is the system under test and, especially, non first-order systems. For second- or high-order systems, the low-frequency poles introduce low-frequency components into the system output signal. When the frequency of these components is lower than the bin frequency of the FFT, they leak over the frequency spectrum, and the FFT cannot represent them. This catastrophic leakage can be prevented using either a high-pass filter or the windowing technique. As it does not require additional hardware, the windowing technique is commonly used.

**Figure 10:**
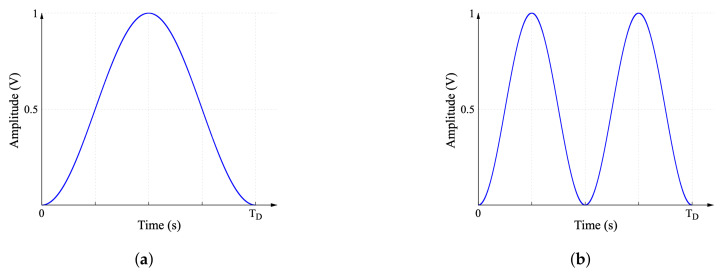
(**a**) Typical Hann window and (**b**) double-frequency Hann window.

The windowing technique is applied by multiplying a signal in the time domain with a specific window function. This operation is known as modulation. Single-tone window functions are ideal for this work as a multi-tone window can distort the frequency components of interest. The Hann window is a popular single-tone window, and it is defined as:

(17) $$W\left(t\right)=\frac{1}{2}\left(1-\cos\left(2\pi Ft\right)\right),$$

where *F* represents the width of the window, and $T_{D}=1/F$. The Hann window is shown in Figure 10a. Though it is useful, the risk of introducing distortion in hardware implementation is high. A double-frequency Hann window is proposed, which also represents a single-tone window, but strategically shifts the frequency components that distort the signal to a location where the risk of distortion is lower than the risk found when a regular Hann window is used. The proposed double-frequency Hann window is shown in Figure 10b and is defined as:

(18) $$W_{DF}\left(t\right)=\frac{1}{2}\left(1-\cos\left(4\pi Ft\right)\right),$$

The effect of the Hann and double-frequency Hann windows over the signal processing are explained by applying them to a single-tone function and are given by:

(19) $$W\left(t\right)\sin\left(\Omega t\right)=\frac{1}{2}\left(\sin\left(\Omega t\right)-\cos\left(2\pi Ft\right)\sin\left(\Omega t\right)\right)$$

(20) $$W_{DF}\left(t\right)\sin\left(\Omega t\right)=\frac{1}{2}\left(\sin\left(\Omega t\right)-\cos\left(4\pi ft\right)\sin\left(\Omega t\right)\right)$$

Observe that the resulting signals have two components: the original signal and the original signal modulated with the single-tone cosine function. According to the modulation theorem, the FFTs of these modulated components are given by:

(21) $$F\left\{x\left(t\right)\cos\left(2\pi Ft\right)\right\}=\frac{1}{2}X(j\left(\Omega \pm 2\pi F\right)$$

(22) $$F\left\{x\left(t\right)\cos\left(4\pi Ft\right)\right\}=\frac{1}{2}X(j\left(\Omega \pm 4\pi F\right)$$

**Figure 11:**
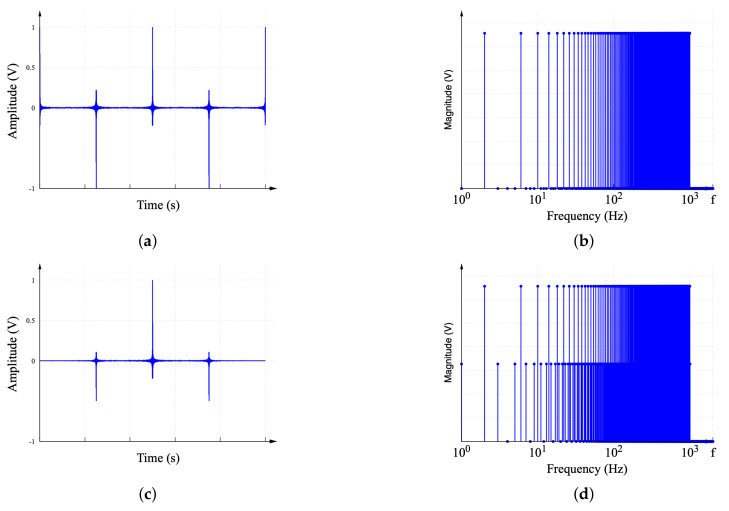

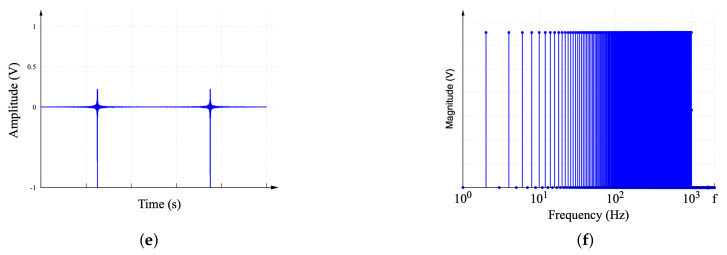
Use of window functions. (**a**) No-windowed MDF $R_{1_{1000}}\left(t\right)$. (**b**) Frequency spectrum of the MDF $R_{1_{1000}}\left(j\omega \right)$ with no window. (**c**) MDF $R_{1_{1000}}\left(t\right)$ using Hann window. (**d**) DFT of MDF $R_{1_{1000}}\left(j\omega \right)$ with Hann window. (**e**) MDF $R_{1_{1000}}\left(t\right)$ using double-frequency Hann window. (**f**) DFT of MDF $R_{1_{1000}}\left(j\omega \right)$ with Hann window.

When a signal is modulated by a window function, the frequency spectrum of the resulting signal, which contains the original signal, and two replicas of this component neighboring the original frequency are observed. Similar replicas are observed when these two window functions are used with the proposed MDF. Figure 11 shows the characteristics of the MDF windowed with the Hann window and the double-frequency Hann window. The no-windowed signal and frequency spectrum are shown, respectively, in Figure 11a,b for comparison. Figure 11c corresponds to the function $R_{1_{1000}}\left(t\right)$ windowed with the Hann window, the frequency spectrum. Meanwhile, Figure 11d shows the spectral content introduced by the windowing process. Observe that the frequency components introduced are one component before and after the frequency component of interest. This is not suitable in a practical implementation as waveform generator instruments cannot generate ideal sine signals with an accurate frequency. Therefore, the zero-magnitude components next to the components of interest should be reserved for waveform generator frequency tolerances. Hence, the use of the Hann window is only convenient in simulation and cannot be used in a practical implementation.

The double-frequency Hann window shown in Figure 11e can be used for practical implementation. This corresponds to the function $R_{1_{1000}}\left(t\right)$ windowed with a double-frequency Hann window. Its frequency spectrum is shown in Figure 11f. Observe that the spaces dedicated to compensating for waveform generator inaccuracies are unoccupied, and the intermediate components between the two frequency components of interest are occupied by the interference produced by the windowing process. These components are the product of interference and can be discarded as a zero-magnitude frequency.

## 4. Implementation and Results

**Figure 12:**
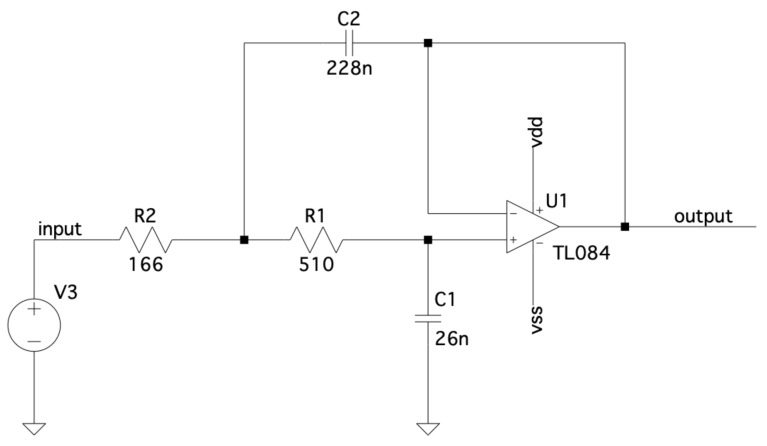
Chebyshev low-pass filter as device under test (DUT).

The proposed frequency response technique using MDF is verified by implementing a second-order Chebyshev low-pass filter as the device under test (DUT). Figure 12 shows the DUT with opamp TL084; resistors R1 and R2; and capacitors C1 and C2. The DUT has a cutoff frequency of 10 kHz, and the frequency response is measured in the 10 Hz to 10 MHz range.

**Figure 13:**
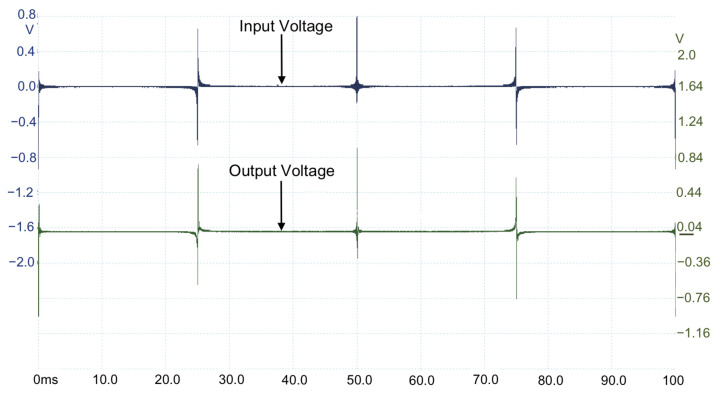
Input and output voltages measured on hardware implementation of $R_{2_{N}}\left(t\right)$ (1–10 KHz).

**Figure 14:**
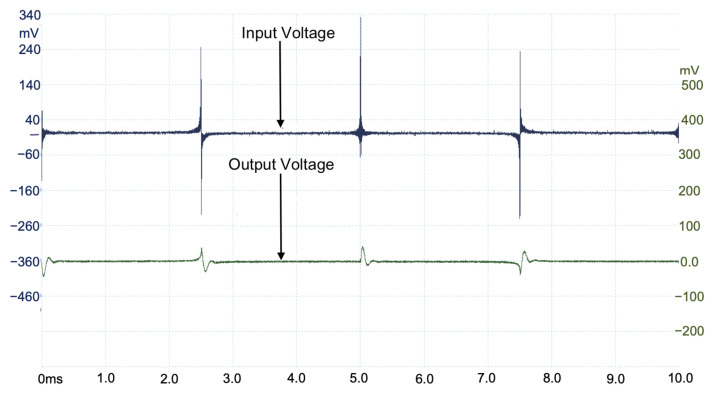
Input and output voltages measured on hardware implementation of $R_{3_{N}}\left(t\right)$ (10–100 KHz).

**Figure 15:**
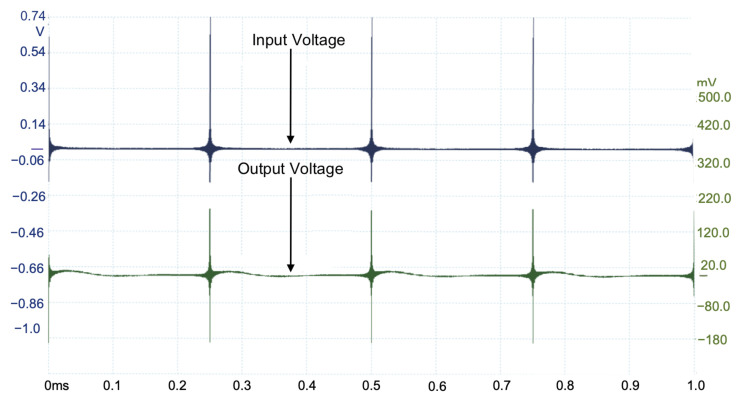
Input and output voltages measured on hardware implementation of $R_{3_{N}}\left(t\right)$ (100 kHz–1 MHz).

The proposed MDF with the spectrum segmentation process and the windowing technique are used as a voltage excitation for the DUT. The six MDFs are configured to measure different spectrum segments. The spectral content of each MDF is configured such that the 900 points/decade resolution is achieved. The six measurements are processed using MATLAB or Octave software to obtain six spectrum segments and are used to integrate the system frequency response in the 10 Hz to 10 MHz range. The second, third, and fourth components of the proposed MDF, corresponding to the 1–10 kHz, 10–100 kHz, and 100–1000 kHz segments, are shown in Figure 13, Figure 14, and Figure 15, respectively. A total of 24 measurements are taken for completing the frequency response.

The proposed double-frequency Hann window is applied to the input and output voltage signals during post-processing, and the FFT of these windowed signals is computed. Assuming that the FFT of the input segment signal is $V_{i}\left(j\omega \right)$, and the FFT of the output segment signal is $V_{o}\left(j\omega \right)$, the magnitude and phase spectra segments are given by:

(23) $$\left|T\left(j\omega \right)\right|=20\log_{10}\left(\right|V_{o}\left(j\omega \right)|)-20\log_{10}\left(\right|V_{i}\left(j\omega \right)\left|\right),$$

(24) $$\phi \left(\omega \right)=\mathrm{angle}(V_{o}\left(j\omega )\right)-\mathrm{angle}\left(V_{i}\left(j\omega \right)\right)\frac{180}{\pi },$$

where |T(jω)| and ϕ(ω) are the magnitude and phase spectrum of the corresponding segment. The overall system magnitude and phase from the segment spectra are obtained by interleaving the spectra of the four components of the MDF and generating the system frequency response.

**Figure 16:**
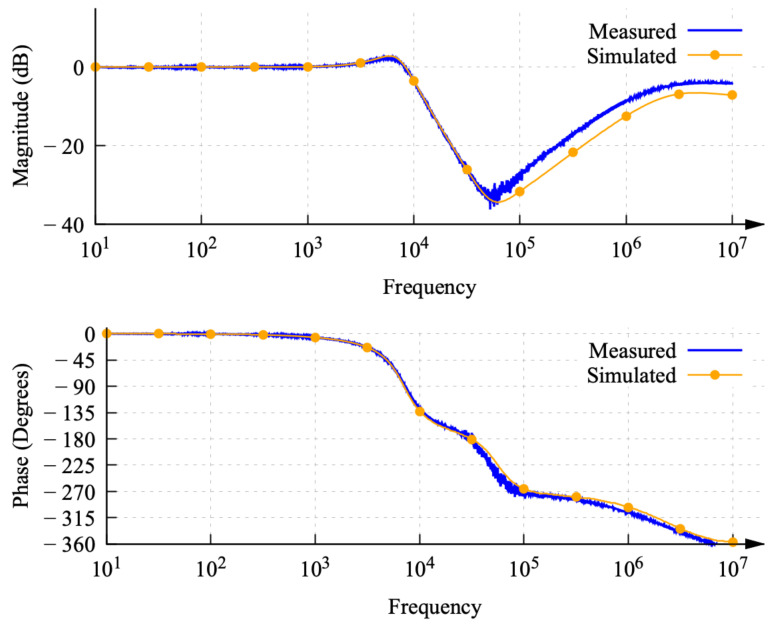
Frequency response of DUT from simulation and measurements showing magnitude and phase plot.

Figure 16 shows the frequency response measured from the DUT using the proposed technique overlapped with the simulation. Observe that the measured and simulated magnitude responses and phase responses shown in Figure 16 are a good match. A deviation is observed in the magnitude response at a high frequency in the range of 50 kHz –10 MHz due to simulation model limitations.

## 5. Conclusions

The frequency response measurement of circuits and systems is proposed using spectrum segmentation, a double Hann window, and a modified Dirichlet function. The proposed method allows the use of low-cost waveform generators, such as AWG instruments, to obtain high-resolution frequency responses with no increased test time. This method is validated with hardware measurements and simulation results. The spectrum segmentation technique reduced the memory size and time requirements to compute the FFT. The proposed technique can be embedded in critical systems, such as electric vehicles, power distribution stations, electronic device design centers, and others in which the frequency response characterization for analysis or fault detection is required for improved reliability. 

## Data Availability

Not applicable.

## References

[1] Roman-Loera A., Veerabathini A., Oropeza L.A.F., Martínez L.A.C., Frias D.M. (2021). Improved Frequency Compensation Technique for Three-Stage Amplifiers. J. Low Power Electron. Appl..

[2] Understanding Challenges in USB Charger Design for Automotive Applications, Application Note 7592. Maxim Integrated Products. https://www.maximintegrated.com/en/design/technical-documents/app-notes/7/7592.html.

[3] Veerabathini A., Roman-Loera A. A Peak Current-Mode Control Boost Converter Model for Stability Analysis: A Design Approach. Proceedings of the 2022 IEEE 65th International Midwest Symposium on Circuits and Systems (MWSCAS).

[4] Yao H., Ning X., Su Y., Liu X., Jin Z. A method for loop-circuit stability analysis. Proceedings of the 2013 International Workshop on Microwave and Millimeter Wave Circuits and System Technology.

[5] Cooman A., Seyfert F., Olivi M., Chevillard S., Baratchart L. (2018). Model-Free Closed-Loop Stability Analysis: A Linear Functional Approach. IEEE Trans. Microw. Theory Tech..

[6] Suárez A., Ramírez F. (2021). Two-Level Stability Analysis of Complex Circuits. IEEE Trans. Microw. Theory Tech..

[7] Ali H., Zheng X., Wu X., Khan S., Saad M. Frequency response measurements of DC-DC buck converter. Proceedings of the 2015 IEEE International Conference on Information and Automation.

[8] Fernández C., Zumel P., Fernández-Herrero A., Sanz M., Lázaro A., Barrado A. Frequency response of switching DC/DC converters from a single simulation in the time domain. Proceedings of the 2011 Twenty-Sixth Annual IEEE Applied Power Electronics Conference and Exposition (APEC).

[9] Ridley R.B. (1991). A new, continuous-time model for current-mode control (power convertors). IEEE Trans. Power Electron..

[10] Faifer M., Piegari L., Rossi M., Toscani S. (2021). An Average Model of DC–DC Step-Up Converter Considering Switching Losses and Parasitic Elements. Energies.

[11] Sharma P., Dhaked D.K., Sharma A.K., Goyal D., Chaturvedi P., Nagar A.K., Purohit S. (2021). Mathematical Modeling and Simulation of DC-DC Converters Using State-Space Approach. Proceedings of Second International Conference on Smart Energy and Communication. Algorithms for Intelligent Systems.

[12] Shen Y., Qin Z., Wang H., Blaabjerg F. (2018). Chapter 3—Modeling and Control of DC-DC Converters. Control of Power Electronic Converters and Systems.

[13] Liu J., Yang P., Lin X., Zhou S. Modeling and simulation of DC/DC converters based on double-loop control. Proceedings of the 2009 3rd International Conference on Power Electronics Systems and Applications (PESA).

[14] Abu-Siada A., Mosaad M.I., Kim D., El-Naggar M.F. (2020). Estimating Power Transformer High Frequency Model Parameters Using Frequency Response Analysis. IEEE Trans. Power Deliv..

[15] Alsuhaibani S., Khan Y., Beroual A., Malik N.H. (2016). Review of Frequency Response Analysis Methods for Power Transformer Diagnostics. Energies.

[16] Al-Ameri S.M., Kamarudin M.S., Yousof M.F.M., Salem A.A., Siada A.A., Mosaad M.I. (2021). Interpretation of Frequency Response Analysis for Fault Detection in Power Transformers. Appl. Sci..

[17] Kornatowski E., Banaszak S. (2020). Frequency Response Quality Index for Assessing the Mechanical Condition of Transformer Windings. Energies.

[18] Behjat V., Vahedi A., Setayeshmehr A., Borsi H., Gockenbach E. (2012). Sweep frequency response analysis for diagnosis of low level short circuit faults on the windings of power transformers: An experimental study. Int. J. Electr. Power Energy Syst..

[19] Sigrist L., Rouco L. (2017). An induction motor model for system frequency response models. Int. Trans. Electr. Energ Syst..

[20] Sant’Ana W.C., Lambert-Torres G., Bonaldi E.L., Gama B.R., Zacarias T.G., Areias I.A.d.S., Arantes D.d.A., Assuncao F.d.O., Campos M.M., Steiner F.M. (2021). Online Frequency Response Analysis of Electric Machinery through an Active Coupling System Based on Power Electronics. Sensors.

[21] Al-Ameri S.M., Alawady A.A., Yousof M.F.M., Kamarudin M.S., Salem A.A., Abu-Siada A., Mosaad M.I. (2022). Application of Frequency Response Analysis Method to Detect Short-Circuit Faults in Three-Phase Induction Motors. Appl. Sci..

[22] Gaviño R. (2010). Introducción a Los Sistemas de Control: Conceptos, Aplicaciones y Simulación con MATLAB.

[23] Ogata K. (2010). Modern Control Engineering.

[24] Golnaraghi F., Kuo B.C. (2009). Automatic Control Systems.

[25] Rajesh S. (2011). A Cmos Mixed-Signal Magnitude and Phase Detector. Master Thesis.

[26] Valdes-Garcia A., Hussien F.A.-L., Silva-Martinez J., Sanchez-Sinencio E. (2006). An Integrated Frequency Response Characterization System With a Digital Interface for Analog Testing. IEEE J.-Solid-State Circuits.

[27] Valdes-Garcia A., Silva-Martinez J., Sanchez-Sinencio E. An on-chip transfer function characterization system for analog built-in testing. Proceedings of the 22nd IEEE VLSI Test Symposium.

[28] Shirazi M., Morroni J., Dolgov A., Zane R., Maksimovic D. (2008). Integration of Frequency Response Measurement Capabilities in Digital Controllers for DC–DC Converters. IEEE Trans. Power Electron..

[29] Miao B., Zane R., Maksimovic D. (2005). System identification of power converters with digital control through cross-correlation methods. IEEE Trans. Power Electron..

[30] Sine Integral Function. https://la.mathworks.com/help/symbolic/sinint.html.

[31] Proakis J.G., Manolakis D.G., Proakis J.G. (1992). Digital Signal Processing: Principles, Algorithms, and Applications.

[32] Oppenheim A.V., Schafer R.W. (2009). Discrete-Time Signal Processing.

[33] Soto M.A., Alem M., Shoaie M.A., Vedadi A., Brès C., Thévenaz L., Schneider T. (2013). Optical sinc-shaped Nyquist pulses of exceptional quality. Nat. Commun..

[34] Da Silva E.P., Borkowski R., Preußler S., Schwartau F., Gaiarin S., Olmedo M.I., Vedadi A., Piels M., Galili M., Guan P. (2016). Combined Optical and Electrical Spectrum Shaping for High-Baud-Rate Nyquist-WDM Transceivers. IEEE Photonics J..

[35] Dirichlet or Periodic Sinc Function. https://la.mathworks.com/help/signal/ref/diric.html.

